# Social conformity persists at least one day in 6-year-old children

**DOI:** 10.1038/srep39588

**Published:** 2016-12-21

**Authors:** Sai Sun, Rongjun Yu

**Affiliations:** 1 School of Psychology, Center for Studies of Psychological Application and Key Laboratory of Mental Health and Cognitive Science of Guangdong Province, South China Normal University, Guangzhou, P.R. China; 2 Department of Psychology, National University of Singapore, Singapore, Singapore

## Abstract

Humans have a tendency to forgo their own attitudes or beliefs in order to better align with the interests of a majority, a behavioral process known as conformity. Social conformity has been widely studied among adults and adolescents, whereas experimental studies on the impact of peer influence among young children have been relatively limited. The current study aims to investigate both short-term and sustained conforming behaviors among children in situations of relatively low social pressure. Forty-one children aged 5 to 6 years rated the attractiveness of 90 faces presented serially followed by witnessing a group rating in the absence of peers. Subsequently, second judgement was made after 30 minutes (Experiment 1). Results show that 6-year-old children tended to conform to their peers when group ratings differed from their own ratings, while younger children did not. In Experiment 2, children were required to make the second judgment one day after exposure to group ratings. Similarly, children aged 6 years exhibited a sustained conformity effect even after one day. Our findings suggest that 6-year-old children spontaneously change their private opinions under implicit social influence from peers.

Humans have a tendency to align their attitudes or behaviors with observed behaviors^1^. They follow the prevailing fashions, succumb to peer influence, and obey the norms established by the group they belong to ref. 2. The act of forgoing one’s own behavioral tendencies, attitudes or beliefs by adopting the behavior of the majority is known as social conformity^3^. Depending on individuals’ intrinsic motivations and disposition toward momentarily adopted behavior changes, conformity may occur in one of two forms^4^. In private conformity, individuals’ attitude may genuinely be swayed for the sake of consistency with what has been recognized as accurate information, resulting in sustained behavioral changes which persist in the absence of the original social influence^4^. In contrast, public compliance refers to outwardly complying with others for the purpose of social affiliation, but inwardly maintaining one’s original attitudes^4^,^5^. Although conformity has potentially deleterious consequences in some circumstance, it helps individuals adapt to uncertain environments, promotes uniformity within group members, and stabilizes diversity among groups, which is crucial for social learning, social cohesion, and cultural heritage^6^,^7^,^8^,^9^.

Conformity is often thought of as a form of group pressure upon the individual, which may come from authorities, adults or peers. Individuals often conform their behavior and opinions to those prevailing in peer group, even when they themselves know better than to agree^2^. Since Asch’s classical perceptual line-judgment paradigm, researchers have studied the phenomenon of social conformity extensively^3^,^10^,^11^,^12^,^13^,^14^,^15^,^16^. In his original experiment, a naive adult participant in a room with seven confederates, who had unanimously agreed about their decisions on the “critical trials”, were instructed to state aloud which comparison line (A, B or C) was most like the target line. They found that about one third (32%) of the naive participants who were placed in this situation went along and conformed to the clearly incorrect majority on the critical trials^17^,^18^. Following Asch’s seminal work, the paradigm of creating an erroneous majority has stimulated numerous inquiries into the nature of conformity among school-aged children, adolescents, adults and elders^3^,^10^,^11^,^12^,^13^,^14^,^16^,^19^. In particular, using the original Asch’s task, Costanzo and Shaw reported a strong curvilinear relation between age and conformity in four participant groups of children and young adults (7–9, 11–13, 15–17, and 19–21 years old)^14^. Moreover, Pasupathi examined individual conformity for judgments of geometric shapes and emotional facial expressions in participants ranging from 18 to 91 years of age, and observed lower rates of social conformity in older participants than in their younger counterparts, especially for judgments of emotional facial expression^16^. These studies have indicated an inverted U-shaped developmental curve for conformity with peers across the span of an individual lifetime^14^,^20^. Besides age difference in conformity, previous studies also revealed a higher rate of conformity among women than men adults^21^,^22^.

Recent findings have shown that even preschoolers are susceptible to the influence of the majority. Using the original Asch’s paradigm, Walker and Andrade found that a large 85% of 3- to 5-year-old children deferred to a majority composed of three of their classmates, whereas 42% conformed in the 6- to 8-year-old group^23^. Using a presentation trick instead of confederates, Hanayama and Mori found that the 6–7 years old minority children who observed slightly different stimuli from the other three children tended to make more errors and conform to the majority^24^. The above two studies indicate a tendency of public conformity among preschool children under social pressure. Moreover, using a modified Asch’s task, Haun and Tomasello found that preschoolers as young as 4-year-old conform more when speaking in public than pointing in private, indicating a discrepancy between public and private conformity^2^. In another study using adults as the confederates, Corriveau and Harris found that children erred by choosing the line that had been indicated by the adult majority^25^. However, when asked to choose the longest line to help a toy rabbit to ford a river by building a bridge, children relied on their own perceptual judgment for their own pragmatic goals. The two studies from Haun *et al*., and Corriveau *et al*. suggest that children only comply with the majority when making an overt and public statement without fully subscribing to the consensus. In the domain of social learning, two more studies demonstrated that children as young as 2 years of age have acquired the behaviors of the majority^26^,^27^. Importantly, children switched much more when the peer demonstrators were still present than when they were absent, suggesting that children adjusted their behavior at least in part for social motivations^26^.

So far, few studies have investigated spontaneous and genuine conformity (a change in private opinion) in children. Thus, the sustained effect of conformity among children remains unclear. Moreover, in Asch’s task, they mainly focused on the judgments with definite answer (e.g. either correct or wrong). For more ambiguous judgments (e.g. facial attractiveness), group opinion may alter the individual opinion to some degree but without ever becoming powerful enough to cause the complete replacement of one attitude with another. Furthermore, previous studies on conformity mainly focused on the influence of explicit social pressure before the presence of a group of people or similar forces, little research has insofar explored spontaneous conformity under low social pressure. Though two studies from Haun *et al*., and Corriveau *et al*. have explored private conformity with “relatively low social pressure”^2^,^25^, the degree of conformity, which is defined as the percentage of false answers in unanimously erroneous conditions, may be exaggerated for the mixture of false response with one’s true own choice. In the present study, using a facial attractiveness judgment task, we aimed to investigate both immediate and sustained conformity behavior among children. We hypothesized that at least immediate conformity would be observed among preschoolers. Moreover, if judgment changes are sustained this is likely to reflect a change in private opinion, whereas if they are transient then this would suggest that only public compliance is involved.

## Behavioral Results

### EXPERIMENT 1

#### Immediate effect of conformity behavior

**Figure 1:**
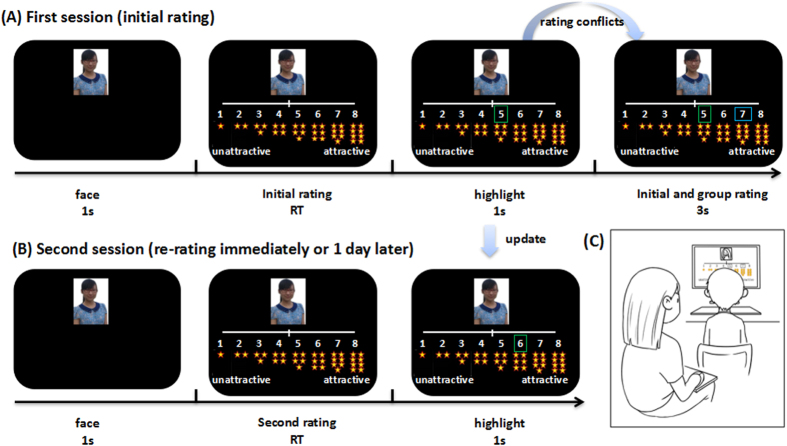
Experimental paradigm. (**A**) Participants were required to rate the attractiveness of the face by reporting to the experimenter. Then they were presented with group ratings. (**B**) After 30 minutes (Experiment 1) or 1 day (Experiment 2), participants were instructed to rate the faces again. (**C**) The experimenter seated behind the child and recorded the ratings.

**Figure 2:**
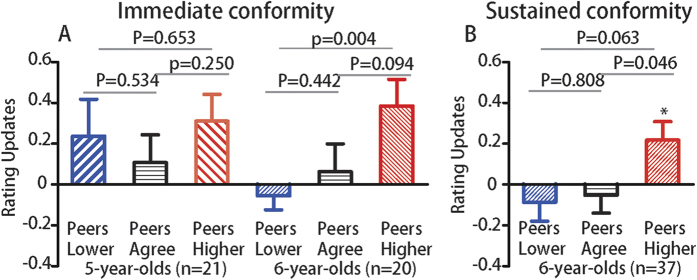
The conformity effect in Experiment 1 and Experiment 2 after controlling for the regression to the mean effect. (**A**) In Experiment 1, the updates (re-rating minus initial rating) after “peers-lower”, “peers-agree”, and “peers-higher” feedback were shown for two groups in immediate re-rating condition, the 6-year-old children tended to conform with their peers, while the younger children did not. (**B**) In Experiment 2, the 6-year-olds children exhibited a sustained conformity effect one day after exposure to group ratings.

Twenty-one 5-year-old and twenty 6-year-old children participated in the study. We asked them to judge the attractiveness of female faces by reporting to the experimenter (Fig. 1A). Then they were presented with group ratings which were equally likely to be above or below children’s ratings by 1, 2 or 3 points. After 30 minutes, participants were instructed to rate the faces again (Fig. 1B). We measured individual conformity by calculating the rating updates between second and initial rating. A two-way repeated-measure ANOVA was conducted with rating updates (re-rating (mean standardized) minus initial rating (mean standardized)) as dependent variable across three conditions (“peers-higher” vs. “peers-agree” vs. “peers-lower”) as within factors, and groups (5-year-olds vs. 6-year-olds) as between factors. We used mean standardized (with a reduction of the mean scores from all participants within each group) initial rating and re-rating scores to reduce strong individual preference and make the results more comparable within each group and between groups. The results revealed a significant main effect of conditions, *F*(2,78) = 3.146, *p* = 0.049, η_p_^2^ = 0.075, and a marginally significant interaction effect between groups and conditions, *F*(2,78) = 3.073, *p* = 0.087, η_p_^2^ = 0.073, suggesting that the degree of conformity varied across two different groups (see Fig. 2A). Two separate repeated-measure ANOVAs with 3 feedback conditions (“peers-higher” vs. “peers-agree” vs. “peers-lower”) as independent variable, and rating updates (after mean-corrected) as dependent variable were performed in 6-year-old and 5-year-old children respectively. We found a significant main effect of feedback among 6-year-old children, *F*(2,38) = 4.22, *p* = 0.022, η_p_^2^ = 0.18, but not among 5-year-old children *F*(2,40) = 0.65, *p* = 0.526, η_p_^2^ = 0.032, suggesting that conflicts from group opinions may lead to subsequent behavioral conformity among 6-year-old children but not 5-year-old children.

Post hoc analysis revealed that 6 year-old participants rated faces as more attractive in the “peers-higher” condition (mean ± *SD*: 0.386 ± 0.579); paired sample t-test, *t*(19) = −1.763, *p* = 0.094, Cohen’s *d* = −0.405, and less attractive but not significant in the “peers-lower” condition (mean ± *SD*: −0.056 ± 0.303); paired sample t-test, *t*(19) = −0.786, *p* = 0.442, *d* = −0.180) compared to “peers-agree” condition (mean ± *SD*: 0.063 ± 0.611) respectively, and also significantly differed from each other (paired sample t-test, *t*(19) = −3.292, *p* = 0.004, *d* = −0.755), even though the faces from “peers-higher” and “peers-lower” conditions had initially been rated as equally attractive (*p* = 0.285). However, no update differences between “peers-higher” (mean ± *SD*: 0.310 ± 0.605) and “peers-lower” (mean ± *SD*: 0.108 ± 0.621) conditions were observed for 5-year-olds (paired sample t-test, *t*(20) = −0.457, *p* = 0.653, *d* = −0.102), and also when compared to “peers-agree” (mean ± *SD*: 0.237 ± 0.829) condition respectively, all *p* values > 0.2.

Besides, we selected faces whose initial rating was 3, 4, 5 or 6, and then compared the rating updates between “peers-lower” and “peers-higher” conditions. We only include these faces with median rating for they are less susceptible to regression to the mean; meanwhile to avoid the instability of limited trial numbers using only faces whose initial ratings were 4 or 5. In particular, the number of trials in each condition with initial rating of 3, 4, 5, 6 was 18.6 ± 8.28 (mean ± *SD*) for “peers-lower”, 15.9 ± 2.55 for “peers-agree”, and 17.15 ± 6.51 for “peers-higher”. For 5-year-olds, the numbers of trials in each condition were comparable to those in 6-year-olds (13.19 ± 5.97 for “peers-lower”, 15.47 ± 3.23 for “peers-agree”, and 12.48 ± 6.48 for “peers-higher”). Similarly, we found a significant difference between “pees-lower” and “peers-higher” among 6-year-olds, *t*(19) = −2.929, *p* = 0.009, *d* = −0.672. Interestingly, no differences were found significantly against “peers-agree” respectively (peers-lower: *t*(19) = −0.585, *p* = 0.565, *d* = −0.134; peers-higher: *t*(19) = −1.293, *p* = 0.211, *d* = −0.297), suggesting that these effects can be out of conflicts from group rating. Moreover, this effect was not found among 5-year-old children, all *p* values > 0.6.

Our results were further confirmed using a linear mixed model. To fit the behavioral judgments of attractiveness, we used the group rating as the fixed effect and initial rating across each subject as the random effect. Two separate models were built among 5-year-olds and 6-year-olds children respectively. Statistical significance of the model was computed by likelihood ratio tests of the full model with the fixed effect of group rating against a null model without the fixed effect of group rating. We found that in the full model, group rating could predict behavioral judgments with a significant regression coefficient (slope; β = 0.076, *p* = 0.006) and intercept (β = 5.35, *p* < 0.001) among 6-year-olds children, and the full model with the fixed effect of group rating significantly outperformed the null model (χ^2^(6) = 7.318, *p* = 0.007), suggesting a significant conformity effect among 6-year-olds children. However, no significant regression effect of group were observed among 5-year-olds children (slope; β = −0.041, *p* = 0.153). Meanwhile, the full model with the fixed effect of group rating did not significantly outperform the null model (χ^2^(6) = 1.940, *p* = 0.164), suggesting that 5-year-olds children subject to group influence but do not exhibit a conformity behavior. To examine whether participants adjusted their ratings symmetrically in the ‘peers-lower’ and ‘peers-higher’ conditions, we added the direction of conflicts as a fixed effect. The group effect was confirmed among 6-year-old children (slope; β = 0.053, *p* = 0.07), but not among 5-year-old children (slope; β = −0.029, *p* = 0.326). However, no significant predictive effect of the direction of conflicts was found (5-year-olds: slope; β = 0.074, *p* = 0.295; 6-year-olds: slope; β = −0.100, *p* = 0.182), suggesting that both positive and negative conflicts with the group modulated second rating to the similar degree (see Fig. 2A).

A further correlation analysis between rating updates and ages were performed among all participants to investigate the developmental process of social conformity among children, however, no significant correlation was found between them (*r* = 0.127, *p* = 0.427). Besides, we tested the gender difference within each group, and found no significant difference among 5-year-olds (independent-sample t-test, *t*(20) = −0.853, *p* = 0.404, *d* = 0.191) and 6-year-olds (independent-sample t-test, *t*(19) = −1.736, *p* = 0.10, *d* = −0.398) respectively. For the sake of completeness, we also reported the response time when making initial judgments. No significant difference were found across different conditions among 5-year-old children (one way repeated-measure ANOVA, *F*(2,40) = 1.278, *p* = 0.285, η_p_^2^ = 0.034) and 6-year-old children (one way repeated-measure ANOVA, *F*(2,38) = 0.092, *p* = 0.912, η_p_^2^ = 0.005) respectively. Taken together, our results suggested a comparable bi-directional conformity based on both positive and negative conflicts with group rating irrespective of gender among 6-year-olds children but not their 5-year-olds counterparts.

## Discussion

Our first experiment addressed whether and to what extent preschoolers conforms to their peers under minimal pressure. We found a significant conformity effect in 6 year-old preschoolers but not in younger 5 year-olds. An equally important aspect of conformity, the question of how firmly or enduringly these behavioral changes are subsequently retained, has not been answered. Conformity may be present in two forms, either as “public compliance” or “private acceptance,” with transient attitude/behavior changes constituting public compliance and persistent ones constituting private conformity. To investigate whether judgment changes based on conformity reflect private acceptance or public compliance, we performed a follow-up study among another 37 children aged 6 years with one day (24 hours) delayed re-rating after exposure to group ratings.

### EXPERIMENT 2

#### Sustained effect of conformity behavior

Compared to “peers-agree” condition (mean ± *SD*: −0.052 ± 0.537) participants rated faces as more attractive in the “peers-higher” condition (mean ± *SD*: 0.218 ± 0.550; paired sample t-test, *t*(36) = −2.068, *p* = 0.046, *d* = −0.345) and as equally attractive compared to the “peers-lower” condition (mean ± *SD*: −0.086 ± 0.567; paired sample t-test, *t*(36) = −0.245, *p* = 0.808, *d* = −0.041) (see Fig. 2B). Further comparison revealed a marginally significant difference between faces from the “peers-higher” and “peers-lower” (paired sample t-test, *t*(36) = 1.919, *p* = 0.063, *d* = 0.319), even though they had initially been rated as equally attractive (*p* = 0.235).

We also selected faces with initial ratings were 3, 4, 5 or 6 (mean ± *SD*: 17.08 ± 5.94 trials for “peers-lower”; 12.08 ± 6.72 trials for “peers-agree”; 18.05 ± 7.47 trials for “peers-higher”), and then compared the rating updates between “peers-lower” and “peers-higher” conditions. Likewise, we found a significant difference between “pees-lower” and “peers-higher” among 6-year-olds (*t*(36) = −4.162, *p* < 0.001, *d* = 0.694). Meanwhile, they both differed significantly against “peers-agree” condition (peers-lower: *t*(36) = −2.115, *p* = 0.041, *d* = 0.353; peers-higher: *t*(36) = −4.162, *p* = 0.052, *d* = 0.335), indicating a one-day sustained conformity effect among 6-year-olds children.

Similarly, a linear mixed model was built to fit the subsequent attractiveness rating with the group rating as the fixed effect and initial rating across each subject as the random effect. Statistical significance of the model was computed by likelihood ratio tests of the full model with the fixed effect of group rating against a null model without the fixed effect of group rating. We found that in the full model, group rating could predict behavioral judgments with a significant regression coefficient (slope; β = 0.048, *p* = 0.015) and intercept (β = 5.44, *p* < 0.001) among 6-year-olds children, and the full model with the fixed effect of group rating significantly outperformed the null model (χ^2^(6) = 5.635, *p* = 0.017), suggesting a significant conformity effect among 6-year-olds children even after one day. When we included the direction of conflicts as a fixed effect, the group effect was confirmed (slope; β = 0.059, *p* = 0.006). Again, no significant predictive effect of the direction of conflicts was found (slope; β = 0.060, *p* = 0.196), suggesting that both positive and negative conflicts with the group modulated second rating in a similar way.

No significant difference of response time were found across three conditions during one day sustained conformity (one way repeated-measure ANOVA, *F*(2,72) = 2.525, *p* = 0.093, η_p_^2^ = 0.112). No significant correlation was found between ages and conformity levels indexed by rating updates, *r* = 0.177, *p* = 0.295. Lastly, no significant gender difference of conformity effect were observed during a one-day sustained conformity (independent sample t-test, *t*(19) = −0.786, *p* = 0.442, *d* = −0.180).

Thus, our findings suggest that the conformity behavior of 6-year-old children is not just a transient form of public compliance, but also entail one-day sustained changes in attitude and belief.

### General Discussion

In our study, we found a significant conformity effect only in 6-year-old children but not in younger 5-year-old children in a context with minimal social pressure. Moreover, we also found that 6-year-old children still conformed to peers even after one day. Our study therefore extends beyond previous studies by showing that 6-year-old children spontaneously conform to others and change their own private opinions even with low social pressure. These findings, if replicated, can enhance our understanding of the socialization process of young children, and especially social conformity among preschoolers.

Social conformity is important for culture acquisition, transmission, and diversity^28^. Conforming to peers may help children adapt to uncertain environment, grasp new skills and maintain social relations^7^. Several studies also investigated conformity in children, albeit their findings are not conclusive. Walker & Andrade found that 85 percentage of 3- to 5-year-old children conform to their peers^23^. Such a high percentage raises the concerns that such young children may not fully understand the task at hand but simply imitate others to satisfactorily provide an answer in most cases. Haun and colleagues found that children as young as 4 years of age conformed more to the majority when responding in public speaking (31.5%) than in private pointing (9.3%)^2^. However, the peer influence was explicitly conveyed by public speaking and oral reporting which is quite different from spontaneous adjustments under low social pressure. A similar study by Corriveau *et al*. also reported that 3 and 4 year-old children sometimes defer to the majority in making simple perceptual judgments^25^. However, they used information provided by adult models rather than by peers. Thus, the reported conformity may be confounded by compliance with authority. Moreover, a recent study demonstrated that children aged 3 to 6 years adjusted their initial judgment based on the characteristics or expertise of their advisors^29^. Similarly, Morgan *et al*. showed that 7-year-old but not younger children were prone to copy the decisions of non-total informants based on the difficulty of each trial and the degree of consensus amongst informants^30^. Both studies suggest an early development of advice taking or social learning among children. Again, the opinions were provided by adults rather than by their peers. In our paradigm, participants were presented with peer ratings individually and privately in the absence of an adult (except for the experimenter) and even peers, which reduced the probability of compliance and minimized the social pressure. Moreover, our manipulation renders it possible to quantitatively measure the degree of behavioral adjustment at a much detailed level, compared with using binary coding (conform or do not conform). Importantly, our results further suggest that spontaneous conformity may develop relatively late (at the age of 6) compared to public compliance among young children.

Notably, one may reason that the failure of 5-year-old children to conform to others may be explained by the underdevelopment of basic cognitive capability (e.g. number sense, memory skills or executive function) or misunderstanding of social task. However, in the current experiment, we mainly investigated the implicit conformity by calculating rating updates between initial rating and second rating during a free-rating task. The task for participants was simply to rate the attractiveness of faces, which does not require high-level memory skills and executive functions, suggesting that the observed behavioral adjustments among 6-year-old children cannot be simply attributed to basic cognitive performance. Another concern is that 5-year-old children may not fully understand the concept of “attractiveness”, especially attractiveness of adults. Dion found that 3-year-olds preschoolers had a discrimination of facial attractiveness of peers, and their judgments were in the same direction as adults’ judgments^31^, suggesting that preschoolers are able to discriminate facial attractiveness of their peers^32^,^33^. Following our previous research^34^, the current study used photos of female adults instead of photos of gender-matched children. Our reliability test has demonstrated a high consistency of attractiveness ratings between children group and the adult samples, suggesting that children in our study can reliably indicate attractiveness of adults’ faces. Nevertheless, future research may use photos of their peers and make the task even easier for young children. Moreover, previous studies have shown that children as young as 4 years old have learned to make numerical magnitude comparisons, such that 8 is greater than 5 or that 6 is smaller than 9^35^,^36^,^37^, suggesting that young children are indeed able to use the rating scale.

In Experiment 1, it remains unclear whether children had changed their private opinions or just exhibited public compliance. In experiment two, with a one-day delayed second rating from initial rating, we found that 6-year-old children also exhibited a sustained conformity behavior at least for one day after exposure to group ratings. Besides, our previous study in adults found that the effect of social conformity can last for no more than three days, reflecting a short-term change in privately held views rather than an instance of transient public compliance^34^. Several functional magnetic resonance imaging (fMRI) studies in adults have demonstrated altered neural processing during conformity in perceptual and reward circuitry^5^,^38^,^39^, suggesting that changes in private attitude, either in perception, evaluation or memory, can occur during conformity. Furthermore, using a transcranial magnetic stimulation manipulation, Klucharev *et al*., found a causal role of the posterior medial frontal cortex engaged in social conformity, indicating an involvement of high-order regulatory function during conformity^40^. In the present study, we provided the first evidence that social conformity effects in 6-year-olds children are enduring, at least over a matter of one day. Such changes in private attitude under social influence may underlie efficient social learning, social consensus, and culture acquisition in children. Whether such sustained effect in children can be extended to 3 days as found in adults remains to be answered^34^.

There are some potential limitations in this study that is worth mentioning. Firstly, though no peers were presented at the time of responding, the presence of the experimenter may create some level of social pressure and lead to demand characteristics. Thus, our results should be interpreted with caution. Future studies should take observation effect into consideration and conduct studies in a double-blind condition, which may partially overcome the demand characteristics, self-selection bias, or placebo effects due to attention, personal contact in an activity. Secondly, the present findings may not apply to the western context, as the participants in our experiments were Han Chinese of East Asian culture. Future studies can further explore the effects of cultural conditions, particularly the dimension of individualism/collectivism, and the effects of individual motives on conformity. Thirdly, it is worth noting that the 6-year-old children (77 ± 7.65 months) in experiment 2 are on average 6 months older than the same group in experiment 1 (71 ± 2.67 months). Future research may further test whether the sustained conformity effect also exists in slightly younger children and whether there are any significant changes on conformity in these few months. Lastly, though our participants were from a similar socioeconomic background, no individual SES data were collected from them or their parents. The variability of SES represented by income, education, and occupation together may still have an influence on children’s socioemotional development and well-being^41^,^42^. In the near future, individual SES data should also be taken into account to the investigation of social conformity behavior among children.

## Methods

### Experiment 1 (Short-term conformity)

#### Participants

Forty-one children from a local municipal kindergarten participated in the experiment. Based on school years and biological age, they were divided into two groups: twenty-one 5-year-olds (12 Males; mean ± *SD*, 58 ± 3.96 months) and twenty 6-year-olds (10 Males; mean ± *SD*, 71 ± 2.67 months). Although socioeconomic background information was not collected for individual participants, the preschools served families from lower to upper middle class demographics. All children in the preschool were invited to participate. Ethnicity information was collected via parental report and with help from the preschool teachers. All children were identified as Han Chinese. The study was approved by the South China Normal University Institutional Review Board and was carried out in accordance with the approved guidelines. All participants were informed of their right to discontinue participation at any time. Parental informed consent was obtained for each child who participated. After completing the experiment, the children were rewarded with chocolate candies for their participation, regardless of their performance.

#### Materials and Procedure

A set of 90 digital photos of front view, Han Chinese female faces with neutral expression was used as stimuli. All photos were downloaded from free Internet sources and were processed in a highly similar photographic style and appearance using Photoshop. These photos had been pre-rated by the participants in previous studies and only those with moderate attraction ratings were selected. The experiment was administered on a Dell laptop, using E-prime (Psychology Software Tools, Inc. Pittsburgh, PA, USA, www.pstnet.com/e-prime) software to control the presentation and timing of stimuli.

In the practice session, participants were first presented with a photograph of a female face (3.5°high, 5.5°wide in visual angle, white against a black background) for 1 second (see Fig. 1). Then the same face and a continuous 8-point Likert scale with a stack of corresponding number (from 1 to 8) of stars below were presented, indicating the attractiveness degree across the range provided. After that, participants were required to rate the attractiveness of the face with the following instructions in Chinese, “Now, we will play a game with tinkle stars. Let’s look at a woman’s face and rate her attractiveness using stars. Stars range from 1, indicating very unattractive, to 8, indicating very attractive. The more attractive you think she is, the more stars you should give to her. How many stars would you like to give to this person”? Then participants were instructed to report the number of stars he/she want to give, and the experimenter (four trained female postgraduates) clicked the corresponding number with a mouse and the selected number was highlighted by a blue rectangle. Participants were informed that the selected number was his/her rating. After that, another rating highlighted by a green rectangle frame and the initial rating highlighted by a blue rectangle were presented simultaneously. Children were told that the selected number with a green rectangle frame was a “group rating” from another peers group in the same grade but different classes of the same kindergarten which had been collected previously (e.g. if the participant was from class 3, we told him/her that the “group rating” was from class 4). We didn’t show participants the photos of their peers to minimize the effects of confounding variables such as attractiveness.

To verify that the participants understood our experiment, the experimenter would point to each face on the screen and repeated for three questions: “How many stars have you given to the person appearing in the screen”? “Do you think the female on the screen is more attractive, attractive or less attractive based on your scores?” and “How many stars did other children give to her”? After answering each item, a corresponding feedback (right or wrong) was given orally by the experimenter. The experimenter was allowed to rephrase the instructions, as many times as needed without making suggestions about how many stars should be given. The practice session comprised 10 trials. We included only participants who answered these questions correctly, indicating that they can successfully distinguish his/her own rating with others’ rating.

In the first experimental session, participants were first presented with a photograph of a female face (3.5°high, 5.5°wide in visual angle, white against a black background) and an 8-point Likert scale with a stack of corresponding number of stars below. Then they were required to indicate their rating choice by oral report. There was no time constraint imposed on the process of making a selection. The experimenter then input the chosen number by clicking the mouse based on the rating provided by the child. The initial rating (highlighted in a blue frame) was presented for 1 second, followed by both an initial rating and a group rating from the peers (highlighted in a green frame) for 3 seconds. The participant was required to pay close attention to both his/her own and others’ ratings. Actual ratings from the alleged peer group were programmed using the following criteria: in 30% of trials, group ratings agreed with the participant’s ratings, whereas in the remaining 70% of trials, group ratings were equally likely to be above or below children’s ratings by 1, 2 or 3 points, using an adaptive algorithm that kept the overall ratio of “*peers-higher*” or “*peers-lower*” ratings approximately equal during the experiment. The deviance from the initial rating is called rating conflict (±1, ±2 or ±3 points). All photographs were randomized across participants and conditions. The whole experiment consisted of two blocks of 90 trials with 2-minutes rest after finishing each block. After the first session, the participant was instructed to rate the faces again without prior warning or knowledge about that they would be asked to do so. No special instructions were given to them except for consideration of stability of behavioral ratings. During the second session, the same faces were presented in a new randomized order without any display of the group ratings. The same experimenter was present throughout the whole experiment.

#### Data analysis

Since the feedback algorithm was constrained such that a trial could be assigned to the “*peers-lower*” condition only when the initial rating of participants was greater than or equal to 4 (allowing the supposed group rating to be at least 3 points lower), a trial could be assigned to the “*peers-higher*” condition only when the initial rating was less than or equal to 5 (allowing the supposed group rating to be at least 3 points higher). Consequently, faces initially rated by participants as more attractive were assigned to the “*peers-lower*” condition disproportionately often, whereas less attractive faces were assigned to the “*peers-higher*” condition disproportionately often. Thus, the rating updates (conformity effect) across the two conditions shown above might partly be explained as “regression to the mean”, which refers to the phenomenon that if a variable is extreme on its first measurement, it will tend to be closer to the average on its second measurement^42^. Thus, the apparent behavioral changes across the “*peers-lower*” and “*peers-higher*” conditions may be confounded with the statistical regression effect.

To solve this problem, we adopted a novel statistical method described by Zaki *et al*.^39^. A subset of faces in which their initial ratings revealed no difference across “*peers-lower*” and “*peers-higher*” conditions were selected among all 5-year-olds and 6-year-olds participants respectively, and then were pooled into further analysis to see whether the subsequent rating updates varied significantly as a function of conditions and/or group. We excluded the faces with initial rating smaller than 10% in the “*peers-higher”* samples and faces with initial rating larger than 85% in the “*peers-lower”* condition for 5-year-olds participants, and faces with initial rating smaller than 16% in the “*peers-higher”* and faces with initial rating larger than 92% in the “*peers-lower”* condition for 6-year-olds participants. The average number of excluded trials for “*peers-lower”* and “*peers-higher”* conditions was 9.38 ± 7.33 (mean ± SD) and 13.05 ± 9.41 for 5-year-olds, 11.50 ± 7.95 and 9.05 ± 6.56 for 6-year-olds. No significant difference of trial numbers was found between “*peers-lower”* and “*peers-higher”* conditions respectively (all *p* values > 0.1). After this exclusion, the difference between “*peers-lower”* and “*peers-higher”* were not significant (5-year-olds: *t*(19) = 1.027, *p* = 0.316, *d* = 0.236; 6-year-olds: *t*(20) = 1.10, *p* = 0.284, *d* = 0.252, respectively). We expected a higher update for the “*peers-higher*” condition but a lower one for the “*peers-lower*” condition during the re-rating session according to conflicts. The Mauchly test was used to assess the validity of the sphericity assumption in ANOVAs. Greenhouse-Geisser corrections were used when sphericity was violated. For the t-test, our results were reported based on the variances assumed equally or not. Alpha level for all tests was.05 two tailed.

### Experiment 2 (Sustained conformity)

#### Participants

Thirty-seven 6-year-olds (14 Males; mean ± *SD*, 77 ± 7.65 months) from another local municipal kindergarten participated in the experiment. The preschools served families from lower to upper middle class demographics. The study was approved by the South China Normal University Institutional Review Board and was carried out in accordance with the approved guidelines. All participants were informed of their right to discontinue participation at any time. Parental informed consent was obtained for each child who participated.

#### Materials and Procedure

The task for Experiment 2 was identical to Experiment 1 except that participants were required to complete the re-rating session about 24 hours later.

#### Data analysis

Likewise, we excluded the faces with initial rating scores 12% smaller from the “*peers-higher”* samples and 84% higher from the “*peers-higher”* samples. The average number of excluded trials for “peers-lower” and “peers-higher” conditions was 7.22 ± 5.70 (mean ± SD) and 11.87 ± 6.70. After this exclusion, the difference between “*peers-lower”* and “*peers-higher”* was not significant (*p* = 0.28).

#### Internal reliability of experimental task within each children group

To test the internal reliability of attractiveness ratings within each group, we first calculated the Cronbach´s alpha using initial rating within each group in study 1. We found that individuals from 5-year-olds (Cronbach’s a = 0.511, Cochran’s Q = 412.086, *p* < 0.001) and 6-year-olds (a = 0.646, Q = 198.071, *p* < 0.001) have highly consistent initial judgments of facial attractiveness. Similar results were found during the immediate second rating (5-year-olds: a = 0.492, Q = 380.676, *p* < 0.001; 6-year-olds: a = 0.620, Q = 210.446, *p* < 0.001). In study 2, we also found high internal consistency for initial rating (a = 0.952, Q = 588.789, P < 0.001) and one-day delayed second rating (a = 0.886, Q = 504.922, P < 0.001). These results suggested a high internal reliability of attractiveness rating within each group.

#### External reliability of experimental task between groups

To test the external reliability of experimental task among children, and make a comparison between children and adults, we also collected data from sixteen adults (7 Males; mean ± *SD*, 20.63 ± 1.82 years) using the same task with immediate second rating except that the adults made responses by using the keyboard. Not surprisingly, we found significant immediate conformity effect in the adults.

To assess the external reliability of ratings, we performed a correlation analysis of initial judgments between groups. Our results revealed a significant positive correlation between 5-year-olds and adults (*r* = 0.56, *p* < 0.001), between 6-year-olds and adults (*r* = 0.59, *p* < 0.001). These results suggested a high consistency of initial attractiveness judgments among children and adults. Similar results were found in study 2. We found significant positive correlations between children and adults for both initial rating (*r* = 0.754, *p* < 0.001) and second rating (*r* = 0.745, *p* < 0.001). These results demonstrated a high consistency of both initial and delayed second attractiveness adjustments among children and adults, and indicated a high external reliability of the experimental task.

## Additional Information

**How to cite this article**: Sun, S. and Yu, R. Social conformity persists at least one day in 6-year-old children. *Sci. Rep.* **6**, 39588; doi: 10.1038/srep39588 (2016).

**Publisher's note:** Springer Nature remains neutral with regard to jurisdictional claims in published maps and institutional affiliations.
